# Unusual presentation of a large glomus tympanicum with a coexisting cholesteatoma

**DOI:** 10.1002/ccr3.3175

**Published:** 2020-07-22

**Authors:** Kartike Gulati

**Affiliations:** ^1^ Otolaryngology‐Head and Neck Surgery Franciscan Health Inc Munster IN USA

**Keywords:** cholesteatoma, glomus, hearing loss, tympanicum

## Abstract

A glomus tympanicum with chronic infectious otorrhea should have a lower threshold for surgical exploration as an occult cholesteatoma may be present in a location difficult to diagnose without performing a tympanomastoidectomy.

## Graphical abstract

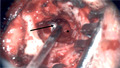

## 1 INTRODUCTION

Glomus tympanicum is a type of benign tumor classified under a group of tumors known as paragangliomas. Such tumors can occur at any site within the body. It is the most common primary neoplasm of the middle ear, and the second most common tumor of the temporal bone.^1^ Most common presentation of a glomus tympanicum is pulsatile tinnitus and conductive hearing loss.^2^ Occasionally symptoms may include otalgia or generalize facial pain.

Although these lesions are histologically benign, they tend to be slow growing, locally destructive, spread along the path of least resistance.^2^

Glomus tympanicum Glasscock‐Jackson classification includes type 1 which is a small mass limited to promontory, type 2 which is tumor completely filling middle ear space, type 3 which is tumor filling middle ear and extending into mastoid, and type 4 which is tumor filling middle ear, extending into mastoid or through tympanic membrane to fill external auditory canal and may extend anterior to carotid.^3^

In this case report, we highlight an unusual and rare presentation of an extensive glomus tympanicum tumor with a coexisting atticoantral cholesteatoma.

## 2 CASE REPORT

**Figure 1:**
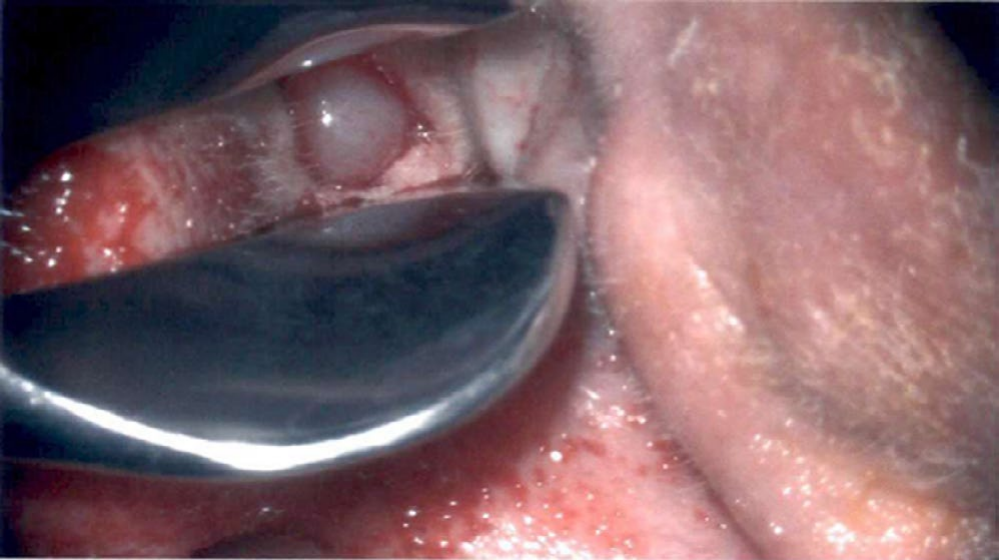
Preoperative view of paraganglioma extending into the external auditory canal

The case report protocol number 1584204‐1, titled Unusual presentation of a large glomus tympanicum with a coexisting cholesteatoma was approved by Franciscan Research Administration institutional review board. A 59‐year‐old male with a past medical history of asthma, hypertension, and ocular migraine presented with a long‐standing right unilateral hearing loss that becomes more apparent about 8 years ago. Hearing loss was nonfluctuating and was profound to the point he could not use a telephone. His right ear had otorrhea ongoing for years that typically was foul smelling and only temporarily resolved with both oral and ototopical antibiotics. Over the past year, he required antimicrobial therapy every 2‐3 months. Patient denies vertigo, headaches, otalgia, and previous ear surgeries. He reported tinnitus that was high‐frequency nonpulsatile in nature. Examination initially demonstrated purulence in the ear canal along with some squamous debris. After debridement of the ear canal and administration of oral and ototopical antimicrobial therapy, further examination demonstrated a polypoid mass completely filling about half of the ear canal representing a glomus tumor. There was no visualization of a tympanic membrane or ossicular chain (Figure 1).

An audiogram was ordered showing right‐sided profound sensorineural hearing loss with speech reception threshold at 95 decibels (dB). A computed tomography (CT) temporal bones without contrast and magnetic resonance imaging (MRI) of brain and internal auditory canal (IAC) with and without contrast were ordered for further evaluation.

**Figure 2:**
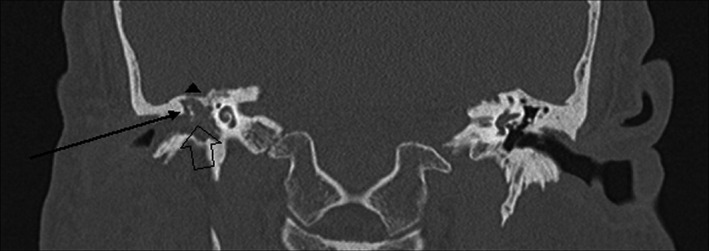
Coronal temporal bone CT view of complete right middle ear and external auditory canal opacification along with evidence of scutum erosion (line arrow), thinning of tegmen tympani (below triangle), and ossicular erosion (arrow)

**Figure 3:**
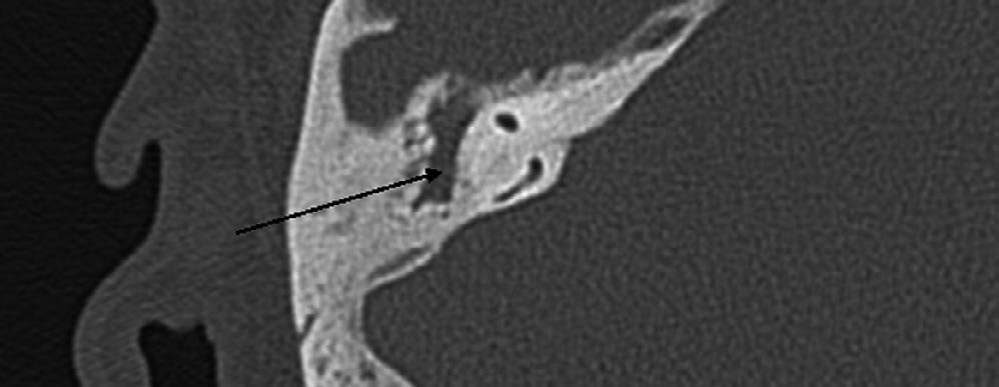
Axial temporal bone CT views of opacification extending into mastoid cavity (see line arrow)

CT temporal bones without contrast showed partial opacification of the right external auditory canal with complete opacification of the right middle ear cavity and hypoplastic right mastoid air cell. Tegmen tympani was grossly intact. Erosive changes of the scutum as well as erosive changes of the right middle ear ossicles including the malleus, incus, and stapes were noted. There was questionable erosion involving the canal wall of the tympanic segment of the right seventh cranial nerve. The right inner ear structures including the cochlea, vestibule, and semicircular canal appeared to be grossly intact (Figure 2 and Figure 3). MRI did not demonstrate intracranial extension, however, confirmed suspicion for a glomus tumor.

**Figure 4:**
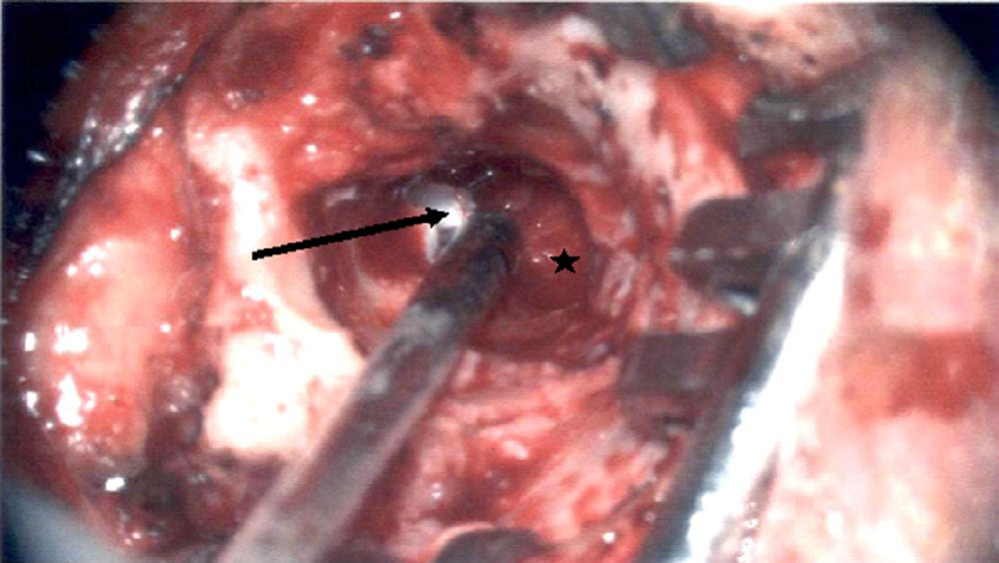
Intraoperative view of atticoantral cholesteatoma (see line arrow) and remaining glomus tumor (see star)

**Figure 5:**
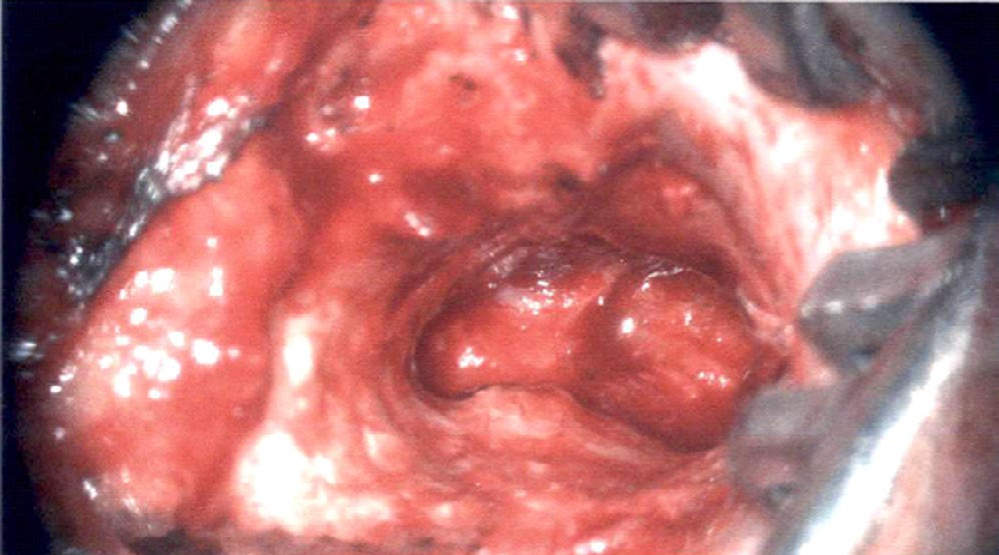
Intraoperative view post glomus and cholesteatoma removal

After reviewing the results, patient agreed to proceed forward with surgery. A right canal wall down tympanomastoidectomy with facial nerve preservation was performed. The glomus tympanicum was completely excised from the ear canal, middle ear, and mastoid. Partial dehiscence was noted along the middle ear segment of the fallopian canal. The ossicular chain was mostly eroded, and the tympanic membrane was completely eroded. Erosion of the scutum was confirmed. The chorda tympani was not found. Adjacent to the glomus tumor was a cholesteatoma in the attic extending into the mastoid antrum, which was completely excised (Figure 4 and Figure 5). The cholesteatoma was visualized only after performing the mastoidectomy. The ossicular chain was not reconstructed due to the level of sensorineural hearing loss. Tragal cartilage was used to obliterate the atticoantral space, and a portion was used in reconstructing the tympanic membrane. Postoperatively, the open cavity healed well. There were no complications.

## 3 DISCUSSION

Coexisting pathology of the temporal bone has been previously reported. One report demonstrates that 11 percent of temporal bones were found to have more than one coexisting pathologic finding such as otitis media, otosclerosis, endolymphatic hydrops, labyrinthitis, and cancer when studied postmortem in an otopathology laboratory.^4^

Our case demonstrated a nonclassical presentation of a Glasscock‐Jackson type 4 glomus tympanicum tumor with a coexisting cholesteatoma. The atypical polypoid appearance of the glomus tympanicum tumor has been previously described where the tumor extending into the external auditory canal mimicked a polyp covered by squamous epithelium.^5^ Our patient did not present with pulsatile tinnitus or conductive hearing loss and in fact had a profound sensorineural hearing loss with high‐frequency nonpulsatile tinnitus. This atypical presentation is likely due to the glomus tumor's late diagnosis. It is possible the delay of diagnosis with lack of treatment allowed the glomus to enlarge extending into the ear canal resulting in trapped squamous epithelium causing a cholesteatoma to form. The cholesteatoma likely then contributed to a presentation of chronic infectious otorrhea. The sensorineural hearing loss may be secondary to the long‐standing infection as previously noted in a correlative study.^6^ Another study demonstrated that sensorineural hearing loss in such presentation could be due to a cochlear fistula caused by chronic otitis media with cholesteatoma.^7^ It is likely the patient may have had an improved hearing result if a diagnosis and treatment had occurred prior to the onset of sensorineural hearing loss.

Previous to an Otolaryngology referral, the patient in our case had been repeatedly treated for otitis media due to his ear examination demonstrating otorrhea and symptoms of hearing loss for many years. It is possible the polypoid nature of his ear examination was confused for a bulging tympanic membrane during this time. After treatment, the otorrhea would temporarily improve giving an opportunity for a reliable ear examination upon follow‐up. The delay in diagnosis may have occurred due to the lack of such follow‐up to confirm resolution of otitis media on examination. This demonstrates the importance of a follow‐up examination after treatment versus trusting resolution of nonspecific symptoms such as otorrhea.

The glomus tumor was diagnosed in the office only after temporarily clearing the infectious otorrhea using antibiotics. Cholesteatoma coexisting with a glomus tympanicum has only once been previously reported in literature representing a rare clinical entity.^8^ Similarly, in our case, the cholesteatoma was found intraoperatively during the canal wall down mastoidectomy. Diffusion‐weighted MRI did not assist in differentiating the glomus tumor from the cholesteatoma in this case. The overall size of the glomus was considerably larger than the cholesteatoma likely making this differentiation difficult on imaging.

Cholesteatomas tend to have some similarities with glomus tumors such as being locally destructive, nonmetastasizing, and causing conductive hearing loss. Cholesteatomas unlike glomus tumors tend to present with chronic otorrhea similar to our case. Extensive glomus tympanicum presentation where the neoplasm extends into the external auditory canal with coexisting chronic infectious otorrhea should have a high clinical suspicion for a cholesteatoma.

The primary management of such extensive glomus tumors is surgery. In certain cases, radiosurgery has been performed in patients with high surgical morbidity and mortality. This is due to the fact that glomus tumors are vascular in nature. One retrospective study has shown that stereotactic radiosurgery is an effective upfront option with or without eventual surgical therapy in the management of glomus tumors.^9^ Radiotherapy in our case would not be an option as it is ineffective in halting the growth of a cholesteatoma.

## 4 CONCLUSION

In conclusion, nonclassical presentation of a glomus tympanicum with chronic infectious otorrhea should have a lower threshold for surgical exploration as an occult cholesteatoma may be present in a location difficult to diagnose without performing a mastoidectomy.

## CONFLICT OF INTEREST

None declared.

## AUTHOR CONTRIBUTIONS

Kartike Gulati: performed evaluation and surgical management for the patient mentioned in the manuscript, wrote the manuscript, and took charge of the overall planning and direction.

## ETHICAL APPROVAL STATEMENT

The case report identification number 1584204‐1, titled Unusual presentation of a large glomus tympanicum with a coexisting cholesteatoma was approved by Franciscan Research Administration institutional review board. Written informed consent was obtained from the participant(s) of this study.
